# Wearable Epileptic Seizure Prediction System Based on Machine Learning Techniques Using ECG, PPG and EEG Signals

**DOI:** 10.3390/s22239372

**Published:** 2022-12-01

**Authors:** David Zambrana-Vinaroz, Jose Maria Vicente-Samper, Juliana Manrique-Cordoba, Jose Maria Sabater-Navarro

**Affiliations:** Neuroengineering Biomedical Research Group, Miguel Hernández University of Elche, 03202 Elche, Spain

**Keywords:** ear EEG, ECG, epilepsy, HRV, machine learning, PPG, PTT, outdoors seizure prediction, wearable

## Abstract

Epileptic seizures have a great impact on the quality of life of people who suffer from them and further limit their independence. For this reason, a device that would be able to monitor patients’ health status and warn them for a possible epileptic seizure would improve their quality of life. With this aim, this article proposes the first seizure predictive model based on Ear EEG, ECG and PPG signals obtained by means of a device that can be used in a static and outpatient setting. This device has been tested with epileptic people in a clinical environment. By processing these data and using supervised machine learning techniques, different predictive models capable of classifying the state of the epileptic person into normal, pre-seizure and seizure have been developed. Subsequently, a reduced model based on Boosted Trees has been validated, obtaining a prediction accuracy of 91.5% and a sensitivity of 85.4%. Thus, based on the accuracy of the predictive model obtained, it can potentially serve as a support tool to determine the status epilepticus and prevent a seizure, thereby improving the quality of life of these people.

## 1. Introduction

Epilepsy is a chronic disease with an enormous social and health impact. It is characterized by recurrent seizures, which are brief episodes of involuntary movement that may involve either a part of the body (partial) or the entire body (generalized) [1]. Status epilepticus can be roughly classified as follows: ictal (seizure), preictal (time before the seizure) and interictal (time between seizures) [2]. Worldwide, roughly 50 million people suffer from epilepsy, making it one of the most common neurological disorders [1]. Despite the treatments and interventions available to assist in the management of the disease, a considerable percentage of patients are not yet fully controlled and continue to have epileptic seizures.

Due to the high impact epileptic seizures have on the quality of life of the people who suffer them, customizable technologies and intelligent systems based on biomedical signals specifically designed for seizure detection have been developed [3,4]. However, they still need to improve their reliability and predictive capacity. Some of the most commonly involved biomedical signals for monitoring an epileptic patient with these devices are Electroencephalogram (EEG), Electrocardiogram (ECG) and Photoplethysmography (PPG).

The EEG measures the electrical activity of the brain, and it has become an essential clinical tool to study and diagnose many neurological diseases, i.e., epilepsy [5,6]. For EEG acquisition in everyday outdoor situations, where the acquisition system must be portable and as comfortable as possible, several ear-focused EEG solutions have been developed [7,8,9]. Significant information can be extracted from the EEG signal by applying data mining techniques for feature extraction or with statistical features, i.e., spectral power, entropy, etc. On the other hand, the ECG measures the electrical activity of the heart [10]. This signal can be used to obtain information about health status from the Heart Rate Variability (HRV) parameter. The heart rate is controlled by the autonomic nervous system. It is regulated by modulations of both the sympathetic and parasympathetic systems [11], mainly influenced by the individual’s circadian rhythms in order to maintain the homeostasis [12]. In addition, HRV parameters extracted from the ECG signal can be used as predictors of seizures [13]. Finally, the PPG is a non-invasive optical technique that is used to detect changes in blood volume in the microvascular layer of the tissue. It allows to detect the pulse wave transmitted through the blood vessels. By combining the ECG signal and this signal, the Pulse Transit Time (PTT) parameter can be calculated. The PTT parameter measures the time it takes for the pulse pressure wave to propagate along the arterial tree. It is widely used as an indicator of blood pressure variability [14], as it is inversely proportional to arterial pressure. Since seizure activity can cause variations in blood pressure, this parameter may be of great interest in the study of epileptic seizures [15,16].

Multiple commercial devices are available to monitor seizures. Embrace2^®^ [17] is a smartwatch that is programmed to detect physical movements and autonomous responses, such as changes in temperature, pulse and respiration. It has different built-in sensors: EDA sensor, Gyroscope, Accelerometer, Temperature sensor, etc. It has a built-in event detector, which detects the electrodermal response. A machine learning algorithm is trained to detect seizure measurements and the model is personalized based on its history. There is also another device that is placed in the ear canal (MJN-Seras^®^) [18] capable of recording and monitoring brain activity. It is equipped with artificial intelligence algorithms that calculate the risk of a seizure in real time, indicating it to the patient by means of a color code. EpiWatch^®^ [19] is an app for Apple Watch users that helps monitor epilepsy by tracking seizures and possible triggers, medications and side effects. SmartWatch^®^ [20,21] is a patented, intelligent, non-invasive wristwatch that continuously monitors the user and instantly alerts family members and caregivers of the appearance of abnormal movement patterns similar to those caused by generalized tonic-clonic seizures. EpiCare@Home^®^ [22] builds a bridge between home and the hospital, since objective seizure-related data are collected in the comfort of the user’s home and securely shared with the neurologist. As a result, the neurologist can develop a more personalized treatment plan for the patient.

There are also many research projects in which devices for monitoring and predicting epileptic seizures have been developed. For example, the work developed by Yamakawa et al. [23] proposes a wearable system for the prediction of epileptic seizures by anomaly detection based on machine learning. The system was evaluated in seven patients with epilepsy, resulting in the proposed method having a sensitivity of 85.7% to detect changes in heart rate variability before seizures. Another example is the work developed by Billeci et al. [24], in which a preliminary study on the integration of EEG and ECG for patient-specific seizure prediction is presented. EEG synchronization patterns, time and frequency characteristics, as well as measures of interictal series recurrence quantification (RR) analysis were extracted. A support vector machine (SVM) classifier was then applied to classify the pre-ictal and interictal phases by combining the features extracted from the two signals. In the work developed by Nasseri et al. [25], an adaptively trained deep neural network of short- and long-term memory was developed and trained using a modest number of seizure datasets from wrist-worn devices (accelerometry, blood volume pulse, electrodermal skin activity, heart rate and temperature signals). Pavei et al. [26] present a new methodology for epileptic seizure prediction using HRV signals. Eigen-decomposition of HRV parameter covariance matrices was used to create an input for a support vector machine (SVM) based classifier. Zhou et al. [27] used a convolutional neural network (CNN) based on raw EEG signals instead of manual feature extraction to distinguish ictal, preictal and interictal segments for epileptic seizure detection. Kusmakar [28] proposes a wireless remote monitoring system based on a single wrist-worn accelerometer device, which is sensitive to multiple seizure types and is able to detect seizures of short duration.

After an extensive literature review, no devices have been found that simultaneously measure ECG, PPG and ear-EEG. Consequently, a device [29] capable of recording these signals has been used with epileptic patients in a clinical environment. By processing these data and using supervised machine learning techniques, different predictive models have been developed, which are capable of classifying the state of the epileptic person into normal, pre-seizure and seizure. It is therefore the first predictive model based on Ear EEG, ECG and PPG signals. The aim of knowing the pre-seizure states in advance (∼1 min before a seizure in these models) is to warn epileptic persons that they are going to have a seizure imminently and thus improve the quality of life of these people. In the materials and methods Section, the proposed device is described, as well as the experimental protocol performed in the clinical environment. Subsequently, the processing techniques used to treat the acquired data will be shown. In addition, we show the generation of predictive models using supervised machine learning techniques and their validation to classify status epilepticus into: interictal (normal), pre-ictal (pre-seizure) and ictal (seizure). Finally, the results obtained will be shown and the accuracy of these models will be discussed and compared with similar studies presented by other authors.

## 2. Materials and Methods

**Figure 1:**
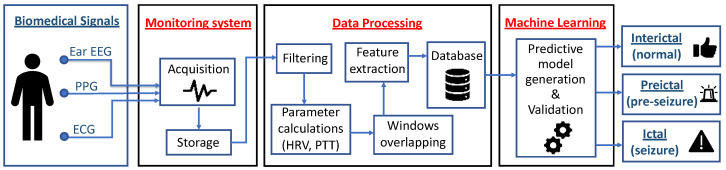
Flow diagram of the methodology.

This Section describes the monitoring system used to acquire the signals studied, as well as the experimental protocol carried out in a clinical setting with epileptic patients. In addition, the processing techniques used to process the acquired data (Filtering, Parameter calculations, Windows overlapping, Feature extraction and Database) are described. Finally, it shows the generation of predictive models using supervised machine learning techniques and their validation to classify the status epilepticus into interictal (normal), preictal (pre-seizure) and ictal (seizure). Figure 1 shows the flow diagram of the methodology.

### 2.1. Monitoring System

**Figure 2:**
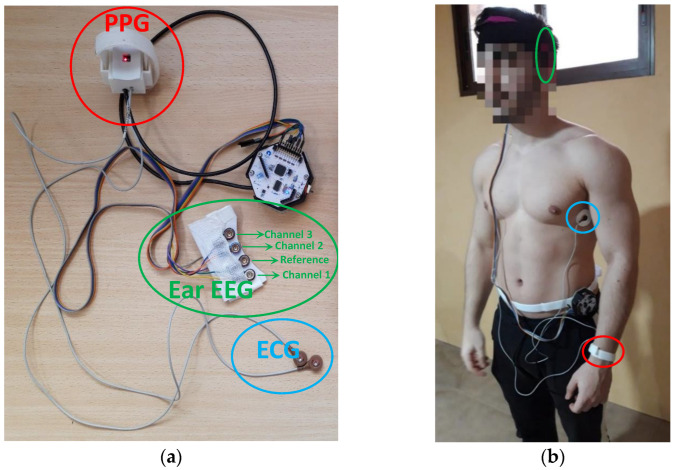
(**a**) Components of the ambulatory monitoring system. Ear EEG is composed of flexible gold cup electrodes (three channels + one reference and noise-canceling); (**b**) Placement of each of the elements that make up the ambulatory monitoring system on the user. Electrodes were placed on both sides of the chest in order to hold the device as tightly as possible and improve comfort.

For the acquisition of the signals studied, a novel monitoring system was developed consisting of different sensors capable of synchronously recording at 250 Hz the electrocardiogram (ECG), photoplethysmogram (PPG) and 3 electroencephalogram (EEG) channels of the ear and storing them for further processing and analysis on a microSD card. Such a system has been extensively described in [29]. This device provides information on health status through the calculation of heart rate variability (HRV), pulse travel time (PTT) and certain characteristics of the ear EEG signal. It has been designed in such a way that it can be used in static and/or ambulatory mode. It has also been validated and compared with other commercial systems with similar results. Figure 2a shows the different components of the monitoring system. On the other hand, Figure 2b shows the final arrangement of the system on a user.

### 2.2. Experimentation Protocol in a Clinical Setting

**Figure 3:**
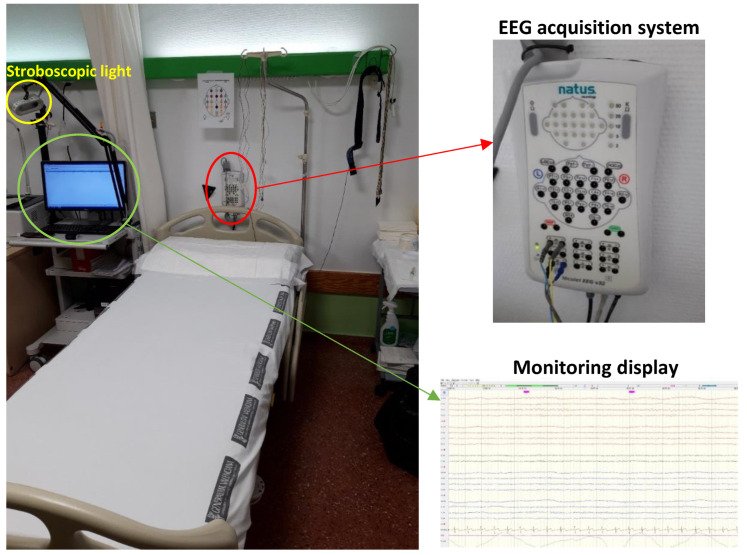
Epilepsy monitoring unit of the Vega Baja Hospital (Spain). This unit has a strobe lamp and an EEG acquisition system (Nicolet EEG v32^®^).

The experimentation was carried out with 10 epileptic volunteers in a clinical setting, exactly in the epilepsy monitoring unit of the Vega Baja Hospital (Figure 3), located in Orihuela (Spain). Table 1 shows information on the epileptic volunteers who participated in the experimentation. The data were recorded by the monitoring system developed in a static way, with the volunteers lying on a bed. At the same time, the neurology team recorded the EEG signal with the Nicolet EEG v32 acquisition system^®^ [30] and an electrode cap 18 EEG signals and then reliably diagnosed the times at which epileptic seizures occurred. Thanks to the information provided by the medical equipment, supervised machine learning techniques can be carried out. It is worth noting that neither system interfered with each other in a negative way in the acquisition of the signals. The experimental procedure lasted 40 min and was designed by the neurology team in order to provoke epileptic seizures and therefore be able to study and diagnose them correctly. In total, 400 min (6.66 h) were recorded. The activities that make up this experimentation are as follows:

1. Open and close eyes (2 min): the user keeps his/her eyes open for 30 s and then closes them for 30 s. Finally, the user repeats it again.
2. Strobe light (6 min): the user is subjected to 12 shots of lights flashing at a frequency of 2, 4, 6, 8, 10, 12, 14, 16, 18, 20, 25 and 30 Hz for 15 s with 15 s pauses between shots.
3. Hyperventilation (1.5 min): The volunteer must hyperventilate, taking 15–20 breaths/min.
4. Rest (2 min).
5. Hyperventilation (1.5 min): The volunteer must hyperventilate, taking 15–20 breaths/min.
6. Rest (2 min).
7. Sleeping (20 min).
8. Waking up (5 min).

### 2.3. Data Processing

Once the experiments were completed, the data stored on the microSD card were analyzed with Matlab software. In order to improve the quality of the signals, filters were implemented. For the PPG signal, a bandpass filter from 0.4 Hz to 3 Hz was used. For the ECG signal, a bandpass filter was applied from 0.4 Hz to 20 Hz and then smoothing. For each of the three ear EEG signals obtained, a bandpass filter from 0.5 Hz to 40 Hz was used. These filtering techniques are extensively discussed in [29].

**Figure 4:**
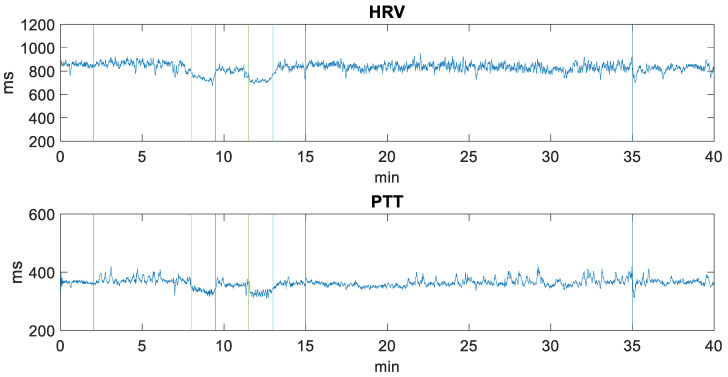
Variation in milliseconds of a volunteer’s HRV and PTT values throughout the experimental procedure (40 min). The vertical-colored lines separate the periods of the different activities developed during the experimentation carried out in a clinical setting with people with epilepsy.

After filtering the ECG and PPG signals, heart rate variability (HRV) and pulse transit time (PTT) were calculated. The HRV parameter is obtained directly from the ECG signal by calculating the time between the R peaks. However, to calculate the PTT parameter, it is necessary to measure the time between the R peak of the ECG signal and the next peak of the PPG signal as indicated in [29,31]. Figure 4 shows the variation in milliseconds of the HRV and PTT values of a volunteer throughout the experimental procedure (40 min). It can be seen how in the hyperventilation period (8–9.5 min and 11.5–13 min) there is a decrease in the values of the HRV and PTT parameters and how at rest their values increase again (9.5–11.5 min and 13–15 min). The reason why it happens in all the cases studied is because hyperventilation causes an increase in heart rate in accordance with [32].

Then, the obtained parameters (HRV and PTT) were divided into time windows with 50% overlapping [33,34] in order to carry out the subsequent feature extraction. Each window is made up of twenty-five HRV and PTT values [35], so the windows are not exactly equal in time (roughly 20 s). However, it is ensured that no window cuts in the middle of an HRV or PTT interval and information is lost. Furthermore, the fact that not all windows have the same duration, being only a few seconds apart, does not have a negative influence. It should be noted that the same time windows discussed above have been used to analyze the ear EEG signal.

Once the data have been divided into windows, the following statistical variables of the HRV and PTT parameters have been calculated for each of these windows [36]: mean, median, mode, standard deviation, variance, absolute deviation, 25th percentile, 75th percentile, interquartile range, kurtosis, skewness, geometric mean and harmonic mean. In addition, the median interval difference, the number of successive interval pairs differing by more than 50 ms in the time domain and the root mean square difference (RMSSD) indicating the activity of the autonomic nervous system [37] were calculated.

Moreover, different features related to the HRV parameters were also analyzed in the frequency domain. Since these features cannot be extracted directly from the HRV values because they are not sampled at equal intervals, the HRV data were interpolated using splines and resampled at equal intervals. For the frequency domain, spectral analysis from the fast Fourier transform (FFT) was used to quantify the density spectrum of very low frequencies (VLF; <0.04 Hz), low frequencies (LF; 0.04–0.15 Hz) and high frequencies (HF; 0.15–0.4 Hz). The LF to HF ratio, which expresses the balance of sympathetic nervous system activity with parasympathetic nervous system activity, was also calculated [38,39]. In total, the following seven features were calculated: VLF, LF, HF, TP (total power), pLF (percentage LF to total power), pHF (percentage HF to total power) and LF/HF ratio.

For each of the 3 filtered ear EEG signals, the following statistical variables were calculated in each of the windows [40,41,42]: mean, median, mode, standard deviation, variance, absolute deviation, 25th percentile, 75th percentile, interquartile range, kurtosis, skewness and harmonic mean. In addition, spectral power [43] and entropy were also calculated, thus generating the following characteristics: mean spectral power, maximum spectral power value, maximum frequency of the maximum spectral power value, median entropy, maximum entropy value and minimum entropy value. Subsequently, each of the 93 characteristics is normalized so that the mean of the set is 0 and the standard deviation is 1, applying equation 1. Where x is each of the entries of the vector of values, $\bar{X\;}$ is the mean and σ is the standard deviation.


(1) $$z=\frac{x-\bar{X\;}}{\sigma }$$

**Figure 5:**
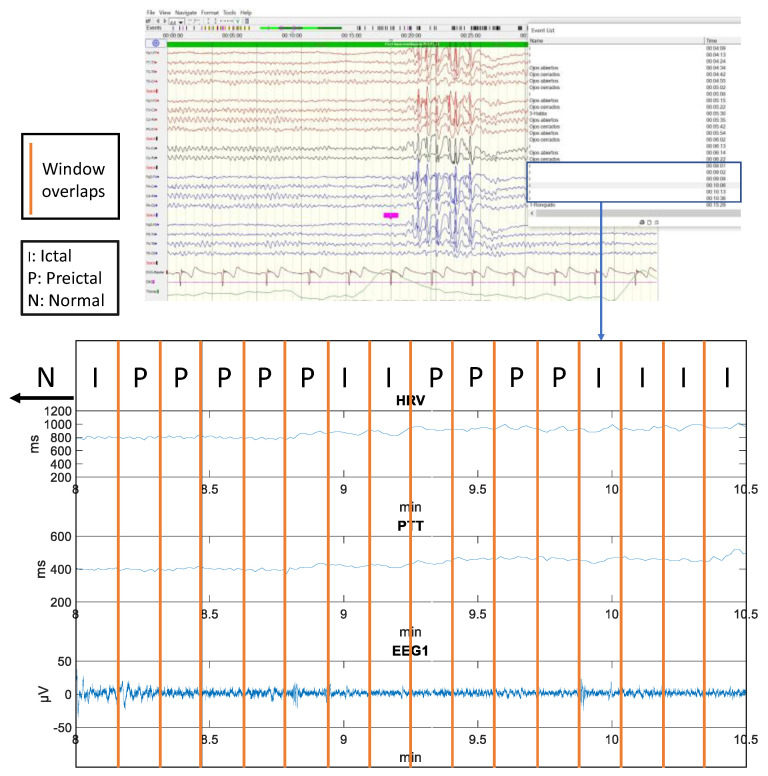
Example of classification of status epilepticus based on the information provided by the medical team.

The normalized data are stored in a spreadsheet for each volunteer. Each entry (window) will be made up of 93 columns (extracted characteristics) and an end column of each window. The purpose of this last column is to be able to know the time instant of each window and to be able to add another extra column, called label, which will indicate according to the diagnosis provided by the neurology team the classification of the epileptic person’s state (N-Normal; P-preictal; I-Ictal). It is noteworthy that the 5 windows (∿1 min) before the onset of an ictal state were classified as preictal as long as no other ictal state appeared [44]. A normal state follows an ictal state unless ictal patterns continue to appear. A preictal state will never appear before a normal state, since the ictal state represents the moments before a seizure. Figure 5 shows an example of how the process of classifying status epilepticus was carried out based on the information provided by the medical team. Once all the labels have been placed, the end column of each window is deleted as it does not provide any information to the predictive models. To make the dataset, all entries from all patients’ spreadsheets are finally pooled and randomly shuffled with the aim of having a heterogeneous distribution of data. In total, the dataset consists of 2153 entries and 94 columns (extracted features and status classification label). Within the 2153 entries: 392 are labelled as I; 505 labelled as P and 1256 labelled as N;

### 2.4. Predictive Model Generation

To carry out the generation of predictive models using supervised Machine Learning techniques, the MATLAB^®^ Classification Learner application has been used [45]. This software allows different predictive models to be generated quickly, providing different tools to evaluate the behavior of the model and search for the best type of classification model, including decision trees, discriminant analysis, support vector machines, logistic regression, nearest neighbors, Naive Bayes, kernel approximation, ensemble, and neural network classification. In addition, it allows the use of principal component analysis (PCA) to reduce the dimensionality of the predictor space [46].

The original dataset has been divided into two parts. The main part will be used for the training of the different predictive models, while the remaining part is reserved for the validation of these models. Specifically, it has been chosen that 70% of the data will be used for training, while the remaining 30% will be used for the validation of the selected predictive model.

To carry out the training of the predictive models, the dataset has been constructed by randomly choosing 70% of the classes from the original dataset. However, in this way the dataset is unbalanced, as the amount of data from the normal class is much more than the rest. Consequently, the generated models have a poor predictive performance, especially for the minority class (ictal). Therefore, the random sub-sampling (RUS) algorithm [47] has been applied to the majority (normal) class. This algorithm is based on selecting examples from the majority class and removing them from the training dataset to balance the data so that all classes have approximately the same amount of data. Finally, the generated dataset has 1000 instances. Specifically, this dataset consists of the following number of classes: 275 seizure; 354 Pre-seizure and 371 normal.

During training, a second division is performed on the aforementioned training dataset. The aim of this new split is to obtain a set used to train the model and a secondary set used to validate the model between training iterations. In this case, the cross validation “k-fold” has been used, with a value of k equal to 5 [48].

**Figure 6:**
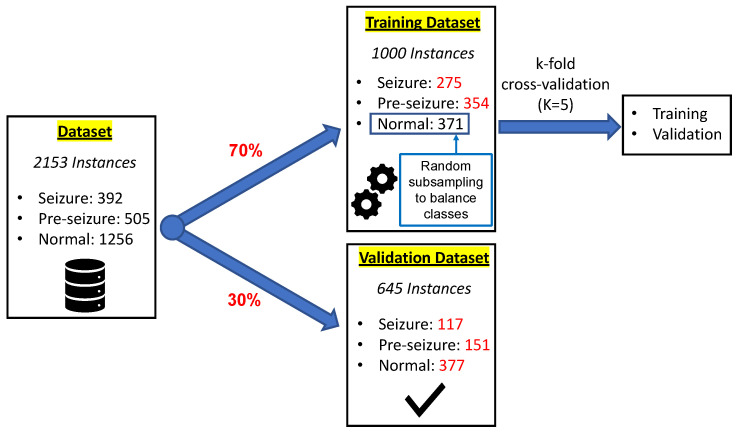
Schematic of the composition of the dataset used in the training and validation of the models.

As for the validation of the selected model, a dataset of 645 instances has been used. Specifically, this dataset is made up of the following number of classes: 117 seizure; 151 pre-seizure and 377 normal. It has been constructed by randomly choosing 30% of the classes of the original dataset and therefore retains the same proportion of the number of labels as the original dataset. Figure 6 shows the schematic of the composition of the datasets used in the training and validation of the models.

## 3. Results

The results obtained after training the different Machine Learning algorithms are shown below. Some models that have been optimized, using smaller predictor space, are also presented. In addition, the results of the validation of the best selected models are also shown.

### 3.1. Predictive Model Training

**Figure 7:**
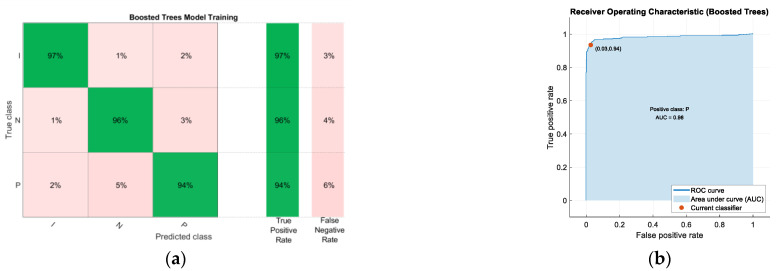
(**a**) Confusion matrix of the Boosted Trees model after training; (**b**) Receiver Operating Characteristic (ROC) curve and its respective Area Under Curve (AUC).

Predictive models have been generated using the whole predictor space and also using only the predictor space corresponding to the characteristics of the ear EEG signal. Table 2 shows the comparison of the results obtained after training the different machine learning algorithms studied, maintaining the default characteristics imposed by the Classification Learner tool. Among all the algorithms analyzed, the model providing the best results is the Boosted Trees model (using the whole predictor space), of the Ensemble Learning type. This model showed a training accuracy of 95.5% after applying Bayesian optimization to obtain the optimal values of the hyperparameters. The confusion matrix after training is shown in Figure 7a. The ROC (Receiver Operating Characteristic) curve and its respective Area Under the Curve (AUC) are included in Figure 7b.

### 3.2. Reduced and Optimized Models

When the model is more complex, the training time increases and the prediction rate decreases. Consequently, to reduce the prediction space and therefore the model, the PCA algorithm has been used. The result of applying this algorithm is displayed in Table 3, where it is shown the variance of original data captured by different number of principal components. In particular, the components have been chosen in such a way that the variance of the original data is maintained at 95%. As a result, 31 components were maintained. The model that provides the best results is Subspace KNN, it has shown 89.9% validation predictions after training.

**Figure 8:**
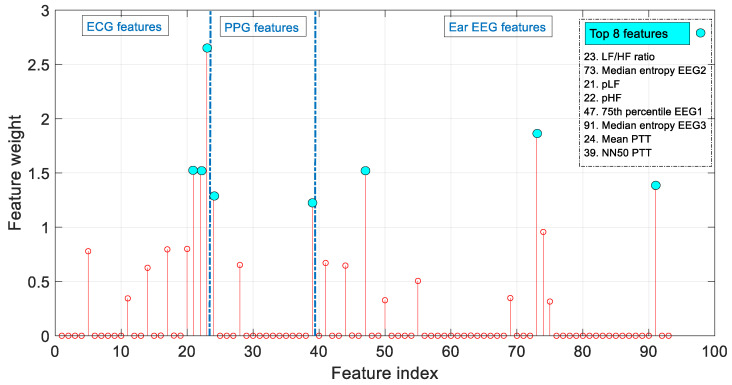
Feature weights as a result of the Neighborhood Component Analysis (NCA).

To assess the importance of the features chosen in the model and to analyze which are the input variables to which the model gives the highest feature weight in the prediction algorithm, the Neighborhood Component Algorithm (NCA) has been used [49]. Figure 8 shows a plot of the feature weights for each of the 93 predictors. In this figure, the 8 predictors with the highest weights (LF/HF ratio, Median entropy EEG2 (EEG channel 2), pLF, pHF, 75th percentile EEG1 (EEG channel 1), Median entropy EEG3 (EEG channel 3), Mean PTT, NN50 PTT) are highlighted. In a system where further optimization is required, the variables that contribute least to the prediction could be excluded, allowing the model to be lightened.

**Figure 9:**
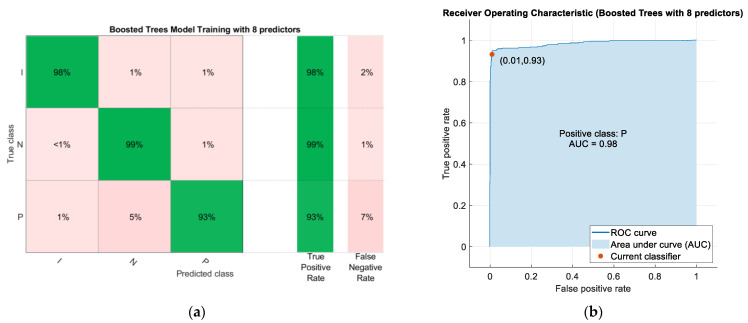
(**a**) Confusion matrix of the Boosted Trees model after training using the 8 predictors with the highest weights after applying the NCA algorithm; (**b**) Receiver Operating Characteristic (ROC) curve and its respective area under the curve (AUC).

It should be noted that by choosing the eight predictors with the greatest weight after applying the NCA algorithm, a model with greater predictive power is obtained. Specifically, the model that provides the best results is Boosted Trees, of the Ensemble Learning type, to which Bayesian optimization has been applied to obtain the optimal values of the hyperparameters. This model has shown 96.5% accuracy after training. Figure 9 shows the confusion matrix of this model, as well as its ROC curve.

### 3.3. Predictive Model Validation

**Figure 10:**
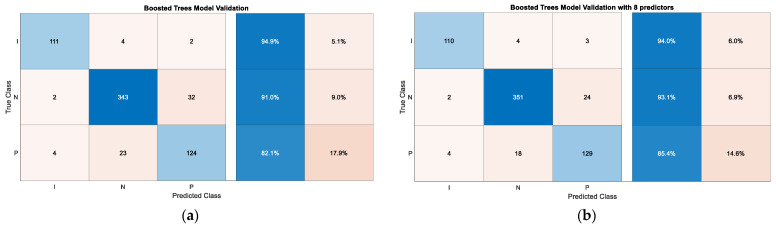
(**a**) Confusion matrix of the Boosted Trees model after validation; (**b**) Confusion matrix of the Boosted Trees model with 8 predictors after validation.

Finally, validation of the Boosted Trees model using the entire predictor space and the Boosted Trees model using only eight predictors was performed with the data set intended for this purpose. The former model was found to have an overall accuracy of 89.61% in predicting status epilepticus against the new data, showing that the preictal class has a sensitivity of 82.1%. Figure 10a shows the confusion matrix as a result of this evaluation.

The second model (using only eight predictors) has an overall accuracy of 91.5% in predicting status epilepticus against the new data, showing that the preictal class has a sensitivity of 85.4%. Figure 10b shows the confusion matrix as a result of this evaluation.

## 4. Discussion

In this Section, the results presented above will be discussed. In addition, a comparison between the proposed device in this work and the proposed in the works of [23,24] is shown.

In view of the results obtained after applying the NCA algorithm, it can be seen that all three signals contribute information to the predictive model. The eight most relevant characteristics are highlighted in Figure 8. The parameters *LF/HF ratio*, *pHF* and *pLF* have a major contribution to the model, since according to [50], during the preictal phase there is a decrease in vagal activity and an increase in sympathetic activity. According to [51], the spectral entropy values for the ictal state are higher compared to the healthy states, thus the *median entropy* parameters (channel 2 and 3) also appear to be relevant. If further optimization is required, the variables that contribute the least to the prediction could be excluded. In this way, the model and the response time would be reduced.

Regarding the results obtained in the training, the Boosted Trees algorithm based on eight predictors was chosen as the model with the highest accuracy (96.5%). The Boosted Trees model based on the whole predictor space was also chosen for having a similar accuracy and being able to compare both models. However, the subspace KNN model obtained after reducing the predictor space using the PCA algorithm presented an accuracy of 89.9% when choosing the first 31 principal components (reducing the predictor space by one third). Since the available data set is not very large, it does not represent an improvement in terms of training time and the Boosted Trees model based on eight predictors presented higher accuracy with an even smaller predictor space.

Figure 7a shows the confusion matrix of the Boosted Trees model after being trained with the full predictor space. A very high true positive rate can be seen. In particular, class I (Ictal) has a true positive rate of 97%, N (normal) also 96% and P (Preictal) 94%. On the other hand, in Figure 7b it can be seen that the area under the curve (AUC) for the Preictal class is very close to 1 (0.98) which means that the results obtained in the evaluation are almost perfect. After validation of the Boosted Trees model based on the whole predictor space, as can be seen in Figure 10a, a lower accuracy than in training was obtained. In particular, class I (Ictal) has a true positive rate of 94.9%, N (normal) also 91.0% and P (Preictal) 82.1%. When validation is performed with an unbalanced data set, it is observed that the algorithm confuses some instances of the normal class with the preictal class and vice versa, as the vast majority of false negatives obtained are due to this reason.

Figure 9a shows the confusion matrix of the Boosted Trees model based on eight predictors after training. It shows a very high rate of true positives. In particular, class I (Ictal) has a true positive rate of 98%, N (normal) also 99% and P (Preictal) 93%. On the other hand, in Figure 9b it can be seen that the area under the curve (AUC) for the Preictal class is very close to 1 (0.98) which means that the results obtained in the evaluation are almost perfect. After the validation of the Boosted Trees model, as can be seen in Figure 10b, the accuracy obtained was lower than in the training. In particular, class I (Ictal) has a true positive rate of 94.0%, N (normal) also 93.1% and P (Preictal) 85.4%. When validation is performed with an unbalanced data set, it is observed that the algorithm confuses some instances of the normal class with the preictal class and vice versa, since the vast majority of false negatives obtained are due to this reason.

In Table 4, a comparison between the proposed device in this work and the ones proposed in [23,24] is shown. This table gathers the most interesting characteristics of this device and compares them to the achievements from different state-of-the-art approaches by other authors (whether a self-developed device has been used to acquire the signals, whether it is wearable, the accuracy of the models, whether it can detect sleep seizures, the algorithm used and the signals that the algorithm is based on). This proposal consists of a device capable of acquiring ECG, PPG and ear EEG signals. From these signals, a Machine Learning model based on Boosted Trees has been developed with a sensitivity of 85.4% of the preictal class. On the other hand, the device developed in [23] only measures ECG and uses multivariate statistical process control (MSPC) with a sensitivity of 85.7%. In [24] no device has been developed, but an SVM model is described based on the ECG and EEG signals obtained with a non-wearable medical device with a sensitivity of 93.3%.

This device and the one proposed in [23] have a very similar sensitivity (∼85%). However, the advantage of our device is that it is based on more signals than just the ECG signal. In an outpatient setting, due to the patient’s movements in daily activities, the signals are significantly worse than those acquired in a clinical setting. The fact that more biomedical signals are acquired makes our device more robust to external disturbances. These external perturbations may not affect equally all acquired signals (bioelectrical signals (ECG and PPG) and optical signal (PPG)). Furthermore, in [23] ictal and interictal data while patients were sleeping were excluded from the analysis, so it does not have the ability to predict seizures while patients are sleeping. However, the generated models in this paper have been developed with data from sleeping patients, as can be seen in the experimental protocol.

In [24], only the implementation of a predictive model is presented, i.e., no wearable device capable of acquiring signals in an ambulatory setting has been developed. The signals have been acquired using medical equipment, which has the ability to eliminate artifacts and thus significantly improve the performance of the predictive models. However, the model presented in this paper and the model in [23] have been implemented with signals acquired with wearable devices. It should be noted that the accuracy of our model could have been higher if the experiment had been performed with volunteers with the same type of epileptic seizures. Given the wearable nature of the used device, it is expected that this model can be tested with new epileptic patients in an outpatient setting.

## 5. Conclusions

A device capable of recording electrocardiogram (ECG), photoplethysmogram (PPG) and ear electroencephalogram (EEG) signals has been used with epileptic patients in a clinical environment. By processing these data and using supervised machine learning techniques, different predictive models capable of classifying the state of the epileptic person into normal, pre-seizure and seizure have been developed.

Although the developed device has been validated only in static conditions in order to compare with the clinical data, the model based on Boosted Trees based on eight predictors has obtained a prediction accuracy of 91.5% using only the data provided by the wearable device. Based on the accuracy of the predictive model, the developed device can potentially serve as a support tool to determine status epilepticus and prevent a seizure, thereby improving the lives of these people. Further advances in this technology can be expected by testing the developed model with epileptic patients in outpatient settings, and thus assessing the predictive capability in the patient’s usual environment, as well as the development of new, more customizable models based on users’ clinical history.

## Figures and Tables

**Table 1:** Information about the volunteers.

Gender	Age (Years)
Female	45
Male	18
Female	32
Male	45
Male	39
Male	42
Female	47
Male	26
Female	35
Male	39

**Table 2:** Comparison of the obtained results after applying different machine learning algorithms using the whole predictor space and only the predictor space corresponding to the EEG signal features of the ear.

Machine Learning Algorithm	Accuracy (%) ECG, PPG, Ear EEG	Accuracy (%) Ear EEG
Fine Tree	92.3	84.2%
Linear discriminant	78.2	78.8%
Kernel Naive Bayes	83.1	78.4%
Linear SVM	75.1	78.3%
Quadratic SVM	83.7	84.0%
Cubic SVM	86.4	84.3%
Cosine KNN	78.2	83.9%
Weighted KNN	80.3	82.1%
Boosted Trees	95.5	86.9%
Bagged Trees	86.2	86.6%
Subspace KNN	93.2	87.9%
RUSBoosted Trees	93.8	87.0%

**Table 3:** Variance of original data captured by different number of principal components.

Number of Principal Components	Explained Variance
10	75%
23	90%
31	95%
72	99.99%
89	100%

**Table 4:** Comparison of the developed model with other status epilepticus classification models.

Characteristics	This Device	Device in [23]	Model in [24]
Device	Yes	Yes	No
Wearable	Yes	Yes	No
Accuracy	85.4%	85.7%	93.3%
Sleep seizure	Yes	No	Yes
Algorithm	Boosted Trees	MSPC	SVM
Signal-based	ECG, PPG, Ear EEG	ECG	EEG, ECG

## Data Availability

Not applicable.
